# A20 Inhibits Intraocular Inflammation in Mice by Regulating the Function of CD4+T Cells and RPE Cells

**DOI:** 10.3389/fimmu.2020.603939

**Published:** 2021-02-04

**Authors:** Jianping Hu, Shenglan Yi, Chaokui Wang, Yiting Zhang, Jihong Tang, Xinyue Huang, Lu Yang, Jinglu Yang, Hong Li

**Affiliations:** ^1^ The First Affiliated Hospital of Chongqing Medical University, Chongqing Key Laboratory of Ophthalmology, Chongqing Eye Institute, Chongqing, China; ^2^ Department of Ophthalmology, The Second Hospital of Lanzhou University, Lanzhou, China

**Keywords:** A20, EAU, Behcet’s disease, blood-retinal-barrier, CD4+T cells

## Abstract

A20 is a negative regulator of inflammation and immunity and plays a role in several autoimmune and inflammatory diseases. Here, we demonstrate that A20 overexpression significantly ameliorates severity of EAU by inhibiting the infiltration of Th1 and Th17 cells, and by protecting integrity of the blood retinal barrier. *In vitro* studies showed that A20 silencing could promote CD4+T cells toward a Th1 and Th17 phenotype. A decreased expression of A20 in CD4+T cells was noticed in active BD patients but not in VKH patients. Furthermore, silencing of A20 in hRPE cells induced the production of IL-6, IL-8, and MCP-1 and downregulated ZO-1 and occludin expression which is mediated by inhibition of MAPK and NF-κB pathways. This study reveals a mechanism by which A20 prevents autoimmune uveitis.

## Introduction

Uveitis is an intraocular inflammatory disease that may cause severe visual impairment (1). Vogt-Koyanagi-Harada (VKH) disease and Behcet’s disease are two common entities in China and Japan (2, 3). Although their etiology remains unclear, recent studies *in vivo* and *in vitro* have indicated that CD4+T cell subsets and the retinal pigment epithelium (RPE) are involved in their pathogenesis.

A20, also called tumor necrosis factor α-induced protein 3(*TNFAIP3*), is directly induced by TNF-α/TNFR signaling, and encoded by the *TNFAIP3* gene (4). It is known as a deubiquitinating enzyme and acts as a negative regulator on inflammation and immunity by inhibiting the NF-κB signaling pathway downstream of TNFR (4). Animal studies showed that A20-deficient mice develop a spontaneous systemic inflammation (4). Mice lacking A20 specifically in B cells (*Tnfaip3^flox/flox^ Cd19-Cre+ mi*ce) spontaneously develop an autoimmune condition similar to SLE, including anti-dsDNA antibodies and glomerular immune-complex deposits (5–7). Mice with an A20 deletion in *CD11c+* DCs (*Tnfaip3^flox/flox^ Cd11c-Cre+ mice*) rapidly develop a significantly disordered immune homeostasis, and showed various phenotypes, such as inflammatory bowel disease, systemic autoimmunity resembling SLE, and multiorgan inflammation (4, 8–10). BMDCs lacking A20 showed an activation phenotype with an increased expression of co-stimulatory molecules (CD80, CD86) and pro-inflammatory cytokines (IL-6, TNF-a, IL-1beta and IL-10) (8, 9). Mice lacking A20 in their macrophages and granulocytes (*Tnfaip3^flox/flox^ Lysm-Cre mice*) developed polyarthritis that was associated with collagen-specific autoantibodies and an increased systemic and local cytokine production (11, 12).

Genetic studies have shown that *TNFAIP3* polymorphisms are associated with several autoimmune diseases such as systemic lupus erythematosus (SLE), inflammatory bowel disease (IBD), multiple sclerosis (MS), rheumatoid arthritis (RA), and psoriasis (8, 13–33). More recently, studies from our group showed that rs9494885 of *TNFAIP3* was associated with VKH and BD in Chinese Han (34, 35). Genetic variants in the *TNFAIP3* region can regulate inflammation and immunity by decreasing the expression and function of A20 (26). Our recent studies showed a decreased expression of A20 in PBMCs from active BD patients (34, 35). All these studies showed that A20 plays a role in the pathogenesis of autoimmune diseases and inflammatory disease, including VKH and BD.

However, it is not clear how A20 exerts its role in their development. In this study, we investigated the role of A20 in the development of uveitis and elucidated possible mechanisms involved.

## Materials and Methods

### Patients

We enrolled active BD patients (n=32), inactive BD patients (n=14), active VKH patients (n=32), inactive VKH patients (n=17), and healthy volunteers (n=46). All subjects were collected in the First affiliated Hospital of Chongqing Medical University and gave written informed consent. BD was diagnosed according to the international nomenclature committee (36). The enrolled BD patients with active intraocular inflammation showed the following ocular manifestations: cells in the anterior chamber, hypopyon, skin lesions, arthritis, oral ulcers, genital ulcers and positive skin allergy reactions. All enrolled activated VKH patients were diagnosed according to the international and Chinese nomenclature committee (37, 38), and showed intraocular inflammation, tinnitus and dysacusis, alopecia, poliosis, and vitiligo. All patients with active VKH and BD were on their first visit in our hospital and had not been treated or stopped taking medicine at least one week before blood sampling. More details of patients are shown in **Tables S2–S4**. All procedures strictly followed the principles of the Helsinki Declaration and were approved by the Ethical Committee of our hospital.

### Mouse Models

B10.RIII mice were obtained from the Jackson Laboratories (Bar Harbor, ME). The full-length mouse *TNFAIP3* cDNA was cloned into a plasmid (**Figure S1A**). Vector genome of AAV2/DJ-GFP and AAV2/DJ-TNFAIP3 (10^8^ copies; Hanbio, Shanghai, China) were injected into the subretinal cavity of mouse eyes as published previously (**Figure S1B**) (39). EAU was induced by injecting 50 µg human IRBP_161–180_ peptide emulsified with complete Freund’s adjuvant as published previously (40). To obtain clinical scores of the EAU model, animals were scored at day 6–20 as described previously (40). Studies conformed to the ARVO statement for the Use of Animals in Ophthalmic and Vision Research. Animal protocols were approved by the Animal Care and Use Committee of the First Affiliated Hospital of Chongqing Medical University.

### Cell Culture

Human primary CD4+ T cells were isolated from PBMCs using mAb-conjugated magnetic microbeads (MiltenyiBiotec, Bergisch Gladbach, Germany). CD4+ T cells were cultured in 24-well plates and were transfected with A20 siRNAs or nonsense sequence using the X-tremeGENE™ siRNA Transfection Reagent (Roche, Germany) according to the manufacturer’s protocol. Subsequently, CD4+T cells were stimulated with anti-human CD3/CD28 (MiltenyiBiotec) for 5 days, then cells were harvested for flow cytometry, and supernatants were collected and frozen.

Human primary retinal pigment epithelium cells (hRPE, purity>90%) were isolated from eyeballs donated by the Chongqing Eye Bank, following a method published earlier (41). The identification of the isolated hRPE was tested by positive immunostaining with RPE65 (**Figure S4**). These cells were seeded into 6-well plates, and were transfected at approximately 75% confluence using A20 siRNAs by Lipofectamine 3000 (Invitrogen, CA, USA) according to the manufacturer’s protocol. Then these cells were cultured to become confluent and starved for 24 h in serum-free medium. Then 5 ng/ml TNF-α or vehicle were added to the medium for 24 h whereafter supernatants and cells were collected. The hRPE cells from passage 3~4 were used.

The concentration of human IL-17, IFN-γ, IL-8, IL-6, and MCP-1 in cultured supernatants was assessed by Duoset ELISA kits (R&D Systems, MN, USA).

### Real-Time PCR

Total RNA was extracted from cells and tissues using the Trizol Reagent (Invitrogen, CA, USA). PrimeScript RT reagent Kit (Takara, Dalian, China) was used to generate cDNA. Real-time PCR was performed with a system (ABI Prism 7500, Applied Biosystems, CA, USA) by using iTaq Universal SYBR Green Supermix (BIO-RAD, CA, USA). The primer sequences are listed in the **Table S1**. Relative mRNA expression was calculated with the 2^-ΔΔct^ method.

### Hematoxylin and Eosin (H&E) Staining

Mouse eyeballs were dissected and fixed with paraformaldehyde. Then, the tissues were washed, dehydrated and embedded in paraffin wax. Serial 6 µm sections were cut through the cornea-optic nerve axis, stained with H&E and scored according to Caspi’s criteria (40).

### Immunofluorescence Staining

Glass slides with hRPE cells and eyeball sections were fixed and blocked. Cells and sections were treated with primary antibody overnight at 4°C, washed and incubated with DyLight conjugated goat anti-mouse IgG antibody (DyLight 488, CA, USA) at room temperature and counterstained with the DAPI reagent.

### Flow Cytometry

For IL-17 and IFN-γ staining, cultured CD4+T cells were stimulated with PMA (50ng/mL) and ionomycin (1g/mL) for 1 h at 37°C, and brefeldin A (Sigma-Aldrich) for another 4 h, then washed, fixed and permeabilized. Fluorescent anti-human CD3-APC, anti-human IL-17A-PE, anti-human IFN-γ-PE-cy7, and anti-human IgG isotype were purchased from eBiosciences.

### Western Blot

Lysates extracted from tissues and cultured cells were separated by SDS-PAGE and proteins were transferred to PVDF membranes. Membranes were incubated after blocking with 5% milk by using the following primary antibodies: anti-A20 (1:1,000, CST), ERK1/2(1:500, Santa Cruz), phospho-ERK1/2 (1:500, Santa Cruz), JNK(1:1,000, Abcam), phospho-JNK (1:1,000, Abcam), P38(1:1,000, Abcam), phospho-P38 (1:1,000, Abcam), P65 (1:1,000, Abcam), phospho- P65 (1:1,000, CST), Ikb-α (Abcam), phospho-Ikb-α (Abcam), ZO-1(1:1,000, Invitrogen), Occludin (1:2,000, Abcam), GAPDH (1:2,000, Abcam) at 4°C overnight. Then, the membranes were washed and incubated with secondary antibodies at room temperature. The membranes were visualized using the Western Bright™ ECL kit (Advansta, CA, USA).

### BRB Integrity

The damage of the BRB was assessed by Evans blue (Sigma-Aldrich, MO, USA). 50 µl of 1% (wt/vol) Evans-blue was injected into the tail veins. The eyeballs were harvested and fixed in fresh 4% (wt/vol) paraformaldehyde for 1 h. Then, the retina was flat-mounted onto clean slides and coverslipped with glycerol.

### Statistical Analysis

Results are shown as mean ± standard deviation (SD). One-way ANOVA and the Kruskal–Wallis test were used to perform multiple group comparisons followed by Dunn’s correction. Paired-samples t-test and Wilcoxon test were used to analyze paired samples. Unpaired t-test and Mann–Whitney U test were used to analyze two independent groups. Statistical analyses were performed with SPSS version 22.0 statistical software (SPSS, Chicago, IL, USA) and GraphPad Prism 7 software (GraphPad Software, Inc, CA). Data were considered significant when p<0.05.

## Results

### Local Overexpression of A20 in AAV Injected Mice

**Figure 1:**
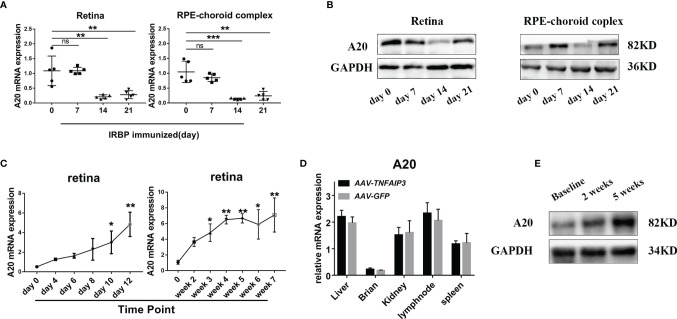
A20 overexpression in the intraocular tissue by *AAV-TNFAIP3* injection. **(A)** Relative mRNA expression of A20 in the retina and choroid-complex from EAU mice in different periods by RT-qPCR (n≥4 per group). **(B)** The protein level of A20 in the retina and choroid-complex from EAU mice in different periods was tested by Western-blot. **(C)** The expression of A20 in the retina after injecting *AAV-TNFAIP3* was tested by RT-qPCR (n=4 per group). **(D)** The expression of A20 in the other organs after injecting *AAV-TNFAIP3* was tested by RT-qPCR (n=4 per group). **(E)** The protein level of A20 in the retina after injecting *AAV-TNFAIP3* was detected by immunoblot. Data are shown as mean ± SD. One-way ANOVA was used for statistical analyis, ns p<0.05, *p<0.05, **p<0.01, ***p<0.001.

We first evaluated the expression of A20 in the retina and RPE-complex of EAU mice on day 0, 7, 14, and 21 following IRBP immunization and found that the expression of A20 was significantly decreased on day 14 (**Figures 1A, B**). Then, we built an adeno-associated virus (AAV) carrying the *TNFAIP3* gene (*AAV*-*TNFAIP3*) which was subretinally injected into the mice. The result showed that A20 mRNA expression was upregulated from day 4 in a time-dependent manner and reached a stable expression peak after 5 weeks (**Figure 1C**). Moreover, A20 was only locally overexpressed in the injected retina, and no A20 overexpression was detected in brain, liver, kidney, lymph node and spleen (**Figure 1D**). At the protein level, A20 was present in the retina of mice injected with *AAV-TNFAIP3* after 2 and 5 weeks (**Figure 1E**).

### A20 Overexpression Attenuates the Severity of EAU Mice and Inhibits Th1 and Th17 Cells Responses

**Figure 2:**
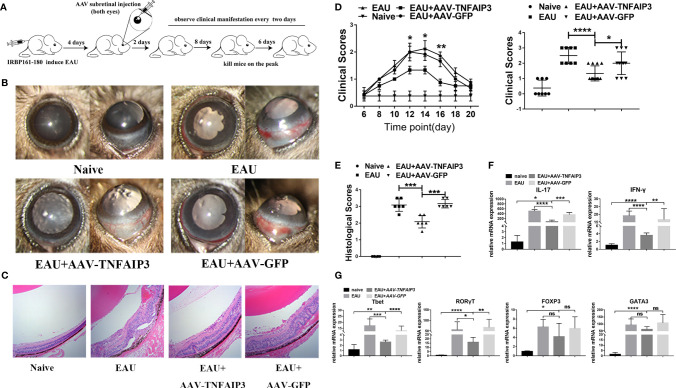
A20 overexpression in early onset suppressed the severity of EAU. **(A)** Both eyes were injected with *AAV-TNFAIP3* or *AAV-GFP*, EAU was induced by IRBP161-180 immunization fourteen days later. **(B)** Representative slit-lamp images of EAU mice at the 14^th^ day after immunization from the frontal and lateral view. **(C)** Representative histological image is shown of eyes harvested at the 14th day after immunization. **(D)** The clinical scores were measured every two days from day 6 after IRBP immunization. The clinical score at the peak of EAU peak (day 14) is shown in the right panel (n≥6 per group, p value was compared between AAV-TNFAIP3 group and AAV-GFP group). **(E)** Histological scores were assessed by hematoxylin and eosin (H&E) staining of paraffin-embedded sections. **(F)** Relative mRNA expression of Th1 and Th17 cytokines IFN-γ and IL-17 by RT-qPCR (n≥4 per group). **(G)** Relative mRNA expression of transcription factors T-bet, RORγT, GATA3, and FOXP3 in CD4+T cells by RT-qPCR (n≥4 per group). Scale bar = 50 μm. Data were shown as mean ± SD. One-way ANOVA was used, ns p>0.05, *p<0.05, **p<0.01, ***p<0.001, ****p<0.0001.

To investigate, the impact of A20 on the uveitis course, we subretinally injected *AAV-TNFAIP3* or *AAV-GFP* (control) 4 days after or 3 weeks before IRBP immunization (**Figure 2A** and **S2A**). The administration of A20 significantly suppressed EAU clinically and histologically (**Figures 2B–E** and **S2B-E**). CD4+ T cells are considered to play essential roles in the process of EAU and we therefore investigated the role of TNFAIP3 on the differentiation of these cells. We found that AAV-TNFAIP3 subretinal injection 3 weeks before IRBP_161-180_ immunization inhibited the expression of the transcription factors T-bet, RORγT, GATA3, and Foxp3 in the retina (**Figure S2G**). However, *AAV-TNFAIP3* subretinal injection 4 days after IRBP_161-180_ immunization only had a minimal effect on T-bet and RORγT mRNA expression (**Figure 2G**). In addition, early or late administration of A20 both inhibited IL-17 and IFN-γ expression in the retina of EAU mice (**Figures 2F** and **S2F**).

### A20 Inhibits IL-17 and IFN-γ Expression in Human CD4+T Cells *In Vitro*

**Figure 3:**
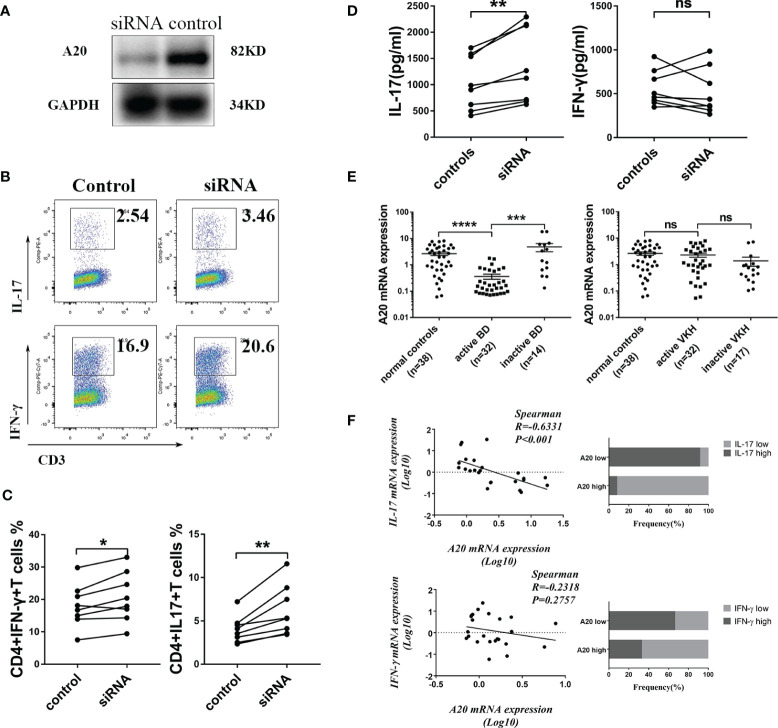
A20 expression was decreased in CD4+T cell from BD patients and A20 knockdown shifted CD4+T cells toward Th1 and Th17 phenotype. **(A)** Immunoblot showing A20 protein inhibition in CD4+T cells treated with A20 siRNA. **(B)** Representative dot plots gated on CD4+ T cells showing IFN-γ and IL-17 expression. **(C)** FACS analysis of the IL-17A expression in activated CD4+ T after A20 was inhibited by siRNA (n=8). **(D)** The production of IL-17 and IFN-γ by A20 knockdown CD4+T cells was tested by ELISA (n=8). **(E)** The mRNA expression of A20 in CD4+T cells from BD patients and VKH patients was tested by RT-qPCR. **(F)** Spearman’s rank correlation coefficient of mRNA expression of A20 vs IL17 and A20 vs IFN-γ (n=24). Data are shown as mean ± SD. One-way ANOVA, Spearman test, Mann Whitney test and paired -T test was used, ns p>0.05, *p<0.05, **p<0.01, ***p<0.001, ****p<0.0001.

To further examine the effect of A20 on CD4+T cells, it was silenced in these cells obtained from healthy donors by siRNA (**Figure 3A**). We found that silencing of A20 significantly shifted CD4+T cells toward a Th1 and Th17 phenotype (**Figures 3B, C**), and induced production of IL-17 in the supernatant of cultured CD4^+^T cells (**Figure 3D**).

Moreover, a decreased A20 expression was observed in CD4+T cells obtained from BD patients but not in active VKH cases (**Figure 3E**). A lower expression of A20 mRNA was found in association with an upregulated expression of IL-17 and IFN-γ mRNA in CD4+T cells from active BD patients (**Figure 3F**). In addition, a normalized expression of A20 in association with the control of the intraocular inflammation was noted in BD patients following treatment with systemic corticosteroids and cyclosporine (**Figure 3E**).

### A20 Induces the Expression of Tight Junction Protein and Inhibits the Expression of Pro-Inflammatory Cytokines in EAU Mice and RPE Cells

**Figure 4:**
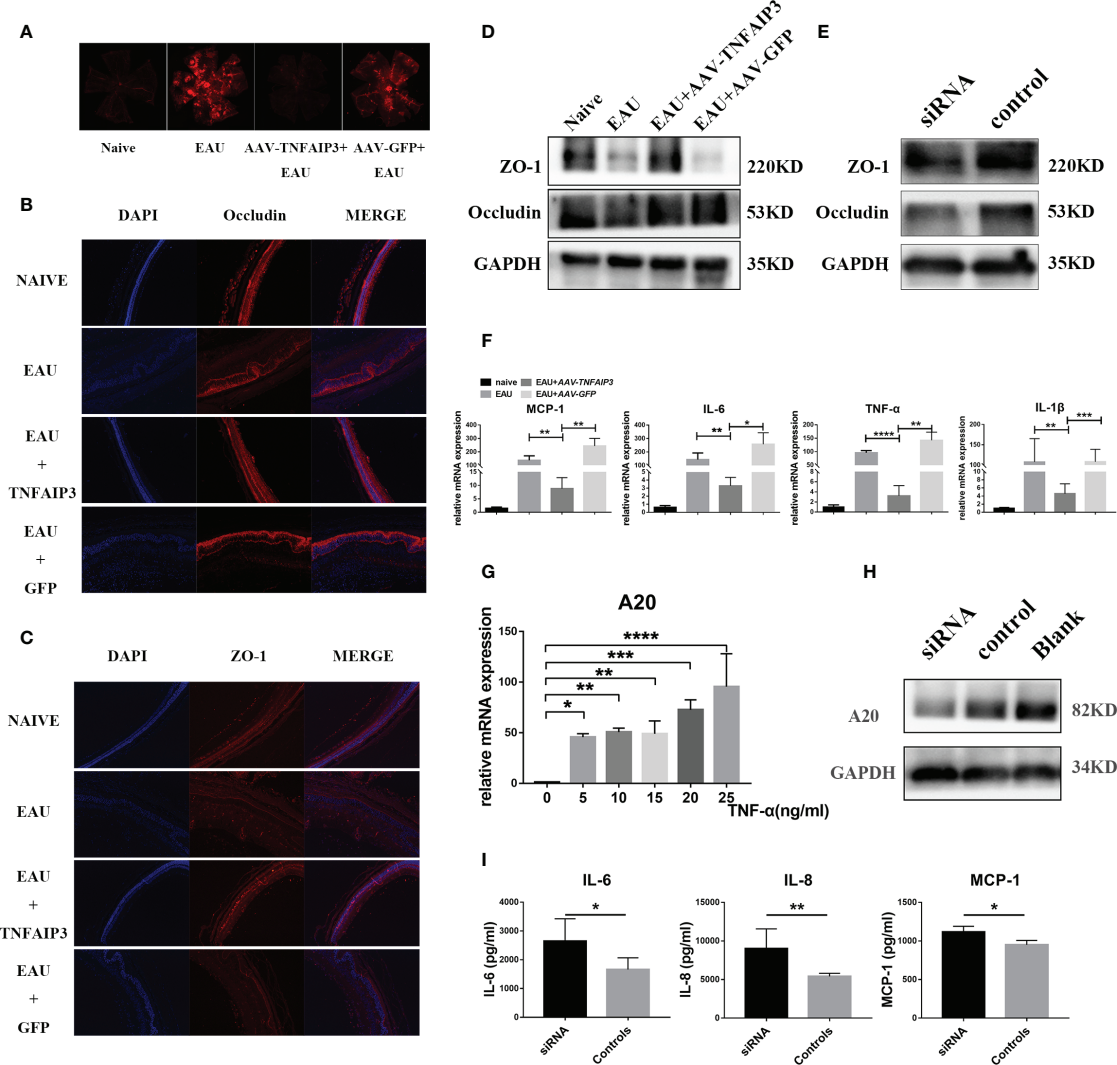
A20 overexpression 14 days prior to IRBP immunization inhibited intraocular inflammation and maintained blood-retinal-barrier (BRB) integrity and tight junction protein expression. BRB integrity of EAU mice was tested by Evans-blue. **(A)** Representative Evans-blue image of the retina from EAU mice in whom A20 overexpression was induced 21 days before IRBP immunization. **(B)** Representative immunostaining of Occludin in retinal sections of EAU mice in whom A20 overexpression was induced 21 days before IRBP immunization. **(C)** Representative Immunostaining of ZO-1 in retinal sections of EAU mice in whom A20 overexpression was induced 21 days before IRBP immunization. **(D)** Western blot tested the expression of ZO-1 and Occludin in the retinal of EAU mice in whom A20 overexpression was induced 21 days before IRBP immunization. **(E)** The expression of ZO-1 and Occludin was tested by Western blot. **(F)** Relative mRNA expression of the cytokines IL-6, MCP-1, IL-1β, and TNF-α by RT-qPCR (n≥4 per group). **(G)** The mRNA expression of A20 in TNF-α-induced stimulation of hRPE cells was tested by RT-qPCR (n=4 per group). **(H)** A20 protein inhibition in hRPE cells by A20 siRNA as tested by immunoblot. **(I)** The expression of IL-6, IL-8, and MCP-1 in culture supernatants was measured by ELISA (n=6 per group). Scale bar = 50 μm. Data are shown as mean ± SD. One-way ANOVA and student’s t test was used, ns p>0.05, *p<0.05, **p<0.01, ***p<0.001, ****p<0.0001.

To further clarify the mechanism whereby A20 prevents the development of intraocular inflammation in EAU mice, Blood-Retina-Barrier (BRB) integrity of EAU mice was assessed by Evans-blue extravasation. We found that *AAV-TNFAIP3* subretinal injection, 3 weeks before IRBP_161-180_ immunization, could inhibit Evans-blue leakage from retinal vessels (**Figure 4A**). In addition, the expression of the tight junction protein ZO-1 in the retina was shown to be induced (**Figures 4B–D**), and the mRNA level of IL-1β, IL-6, TNF-α, and MCP-1 in the retina was reduced by A20 overexpression (**Figure 4F**). We also tested the BRB integrity of EAU mice subretinally injected with *AAV-TNFAIP3* 4 days after IRBP_161-180_ immunization. This did not block inflammation and marked leakage from retinal vessels was observed (**Figure S3**).

In the following *in vitro* experiments, we found that A20 expression in hRPE cells was induced by TNF-α in a dose-dependent manner (**Figure 4G**). A20-siRNA was then used to block A20 expression in hRPE cells stimulated with TNF-α (**Figure 4H**). Blocking A20 resulted in a significantly increased production of IL-6, IL-8, and MCP-1 and reduced tight junction protein ZO-1 and occludin expression by hRPE cells (**Figures 4E, I**).

### A20 Inhibits Inflammation in hRPE Cells and EAU Mice *Via* the MAPK and NF-κb Pathway

**Figure 5:**
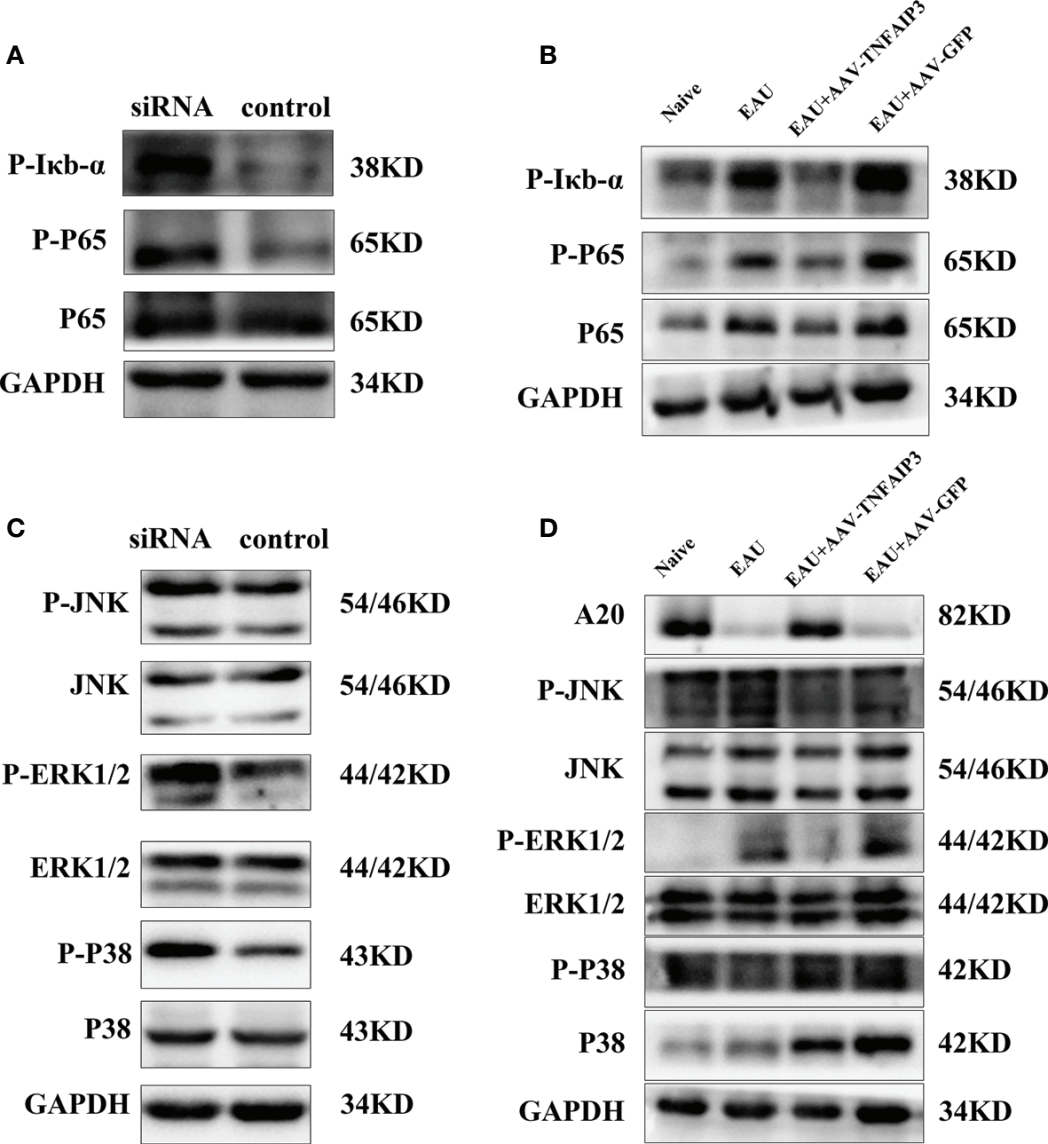
A20 regulates inflammation *via* MAPK and NF-κb pathways *in vitro* and *in vivo*. **(A)** Immunoblot tested the activation of the NF-κb pathway in TNF-α stimulated hRPE cells following A20 siRNA treatment *in vitro*. **(B)** Immunoblot tested the activation of the MAPK pathway in TNF-α stimulated hRPE cells following A20 siRNA treatment. **(C)** Immunoblot analysis testing the activation of the NF-κb pathway in the retina of EAU mice following A20 overexpression *in vivo*. **(D)** Immunoblot analysis testing the activation of the MAPK pathway in the retina of EAU mice following A20 overexpression *in vivo*.

Recent studies have shown that A20 deficiency induced phosphorylation of MAPKs and activated NF-κb signaling (42). Whether A20 has a similar effect on hRPE cells and EAU mice has not yet been shown. A20 knockdown indeed induced phosphorylation of MAPKs and NF-κb activation in hRPE cells (**Figures 5A, B**) confirming the general role of A20 as an endogenous negative regulator of NF- κb signaling.

To investigate whether this mechanism was also effective *in vivo*, we immunized B10RII mice 3 weeks after AAV-TNFAIP3 subretinal injection. The retinal tissue from these EAU mice was harvested 14 days after IRBP immunization. The result showed that the protein level of P38 was reduced by A20 overexpression and that phosphorylation of ERK1/2-MAPK leading to activation of NF-κb was also inhibited (**Figures 5C, D**).

## Discussion

In this study, we demonstrate that the expression of A20 in CD4+T cells from active BD patients is decreased. Silencing of A20 could shift CD4+T cells toward a Th1 and Th17 phenotype. Moreover, we showed for the first time that AAV2/DJ mediated local A20 overexpression significantly inhibited the development of EAU by blocking the activation of the NF-κb and MAPK pathway. A20 expression was not affected in CD4+ cells from VKH uveitis. These data confirm earlier findings from our group where we investigated A20 expression in PBMCs and DCs in uveitis patients (34). The discrepancy between BD and VKH may be due to the fact that VKH is a typical autoimmune disease directed at melanocyte associated antigens, whereas BD is considered as an autoinflammatory disease caused by an exaggerated response against certain exogenous triggers (43, 44).

Autoimmune uveitis is considered to be a T helper cell mediated autoimmune disease (45, 46) and in the study reported here, we found that A20 expression was decreased in CD4+T cells from active BD patients, supporting the hypothesis that A20 plays an important role in the pathogenesis of BD. Genetic studies also showed that various A20/TNFAIP3 polymorphisms confer risk of autoimmune disease, such as rheumatoid arthritis (RA), systemic lupus erythematosus (SLE), systemic sclerosis (SSc), Sjögren’s syndrome(SS), Crohn’s disease, psoriasis, type1 diabetes, and coeliac disease (8, 12–30, 33, 47). The exact biological function of several disease associated A20/TNFAIP3 polymorphisms is not yet clear, but ongoing studies are now addressing this issue. One SNP associated with SLE is present in the coding region of TNFAIP3 and causes a missense mutation that reduces A20 function (26). Some of the risk SNPs near the coding regions of the TNFAIP3 gene have been shown to reduce both A20 mRNA and protein expression (48). In our previous study, a significantly increased prevalence of the TC genotype at rs9494885 was found in BD patients, but the TC genotype had no influence on A20 mRNA expression in PBMCs (47). Since PBMCs include a wide range of cells we may have missed an effect of the rs9494885 SNP variant on a specific cell type. When examining isolated CD4+ cells we found that TC genotype carriers had a reduced A20 mRNA expression compared to other genotypes (data not shown). These results suggest that the TC genotype at rs9494885 confers a risk for BD by reducing A20 mRNA expression in CD4+T cells.

A20 is known to regulate TCR-mediated NF-κb activation and TCR-mediated survival (49–51). Activated CD4+T cells with an A20 deletion die quickly and shift toward a Th1 and Th17 phenotype, due to an A20 control of necroptosis and autophagy (52, 53). These findings are in agreement with our study, where we found that A20 silencing promoted human CD4+T cells toward a Th1 and Th17 phenotype and increased the production of IL-17. To further investigate the impact of A20 on CD4+T cells in the development of uveitis, we investigated the expression of A20, IL-17, and IFN-γ in CD4+T cells from active BD patients. In support of our hypothesis concerning the role of A20 in uveitis pathogenesis, we noticed that the expression of A20 showed an inverse correlation with IL-17 expression. This evidence supports the hypothesis that A20 is a negative immune regulator which acts *via* the inhibition of CD4+T cell activation. This proposed mechanism is supported by our animal study where we showed that an AAV2/DJ mediated local A20 overexpression significantly suppressed the severity of EAU, and inhibited intraocular CD4+T cell infiltration. However, when comparing the effect between an early and late administration of A20 overexpression, it was evident that regulation of A20 is not beneficial when EAU is already ongoing. In clinical practice this might mean that A20 regulation may not directly influence active uveitis, but that it may protect the eye from a relapse of the disease. Further longitudinal studies in patients are needed to address this issue.

It is also possible that A20 can regulate intraocular inflammation by a local protective effect *via* resident cells of the eye. A candidate resident cell type is the retinal pigment epithelium (RPE) layer, which is an important component of the outer blood–retinal barrier (BRB). The RPE is a monolayer of cells connected by tight junction proteins, which contribute to the immune privilege properties of the eye (54). Previous studies showed that A20 is expressed in intestinal epithelial cells (IECs) and can play an important role in protecting intestinal tissue from TNF-α induced inflammation and apoptosis (55, 56). In this model of intestinal inflammation, A20 is thought to play a role as a regulator of the TLR, TNFR, and IL-1R pathway (56, 57), and whereby A20 might also have a significant function during intestinal recovery *via* its potential role in tissue repair (58). RPE cells might play a similar role as the IECs discussed above, which is supported by the finding from our *in vitro* studies where we showed that A20 could reduce TNF-α-induced inflammation in human primary RPE cells. In addition, A20 also prevented RPE cells from TNF-α damage by inducing an increased expression of the tight junction protein ZO-1, both *in vivo* and *in vitro*. As already mentioned above, only an early A20 overexpression prior to EAU induction was able to play a protective role on blood-retinal-barrier (BRB) integrity.

In summary, our study showed that the expression of A20 in CD4+T cells was decreased in active BD patients. We further proved that the expression of A20 was strongly correlated with IL-17 expression in CD4+T cells from active BD patients, and silencing of A20 could shift CD4+T cells toward a Th1 and Th17 phenotype. In addition, A20 induced the expression of tight junction protein and inhibited the expression of pro-inflammatory cytokines by regulating NF-κb and MAPKs pathways in RPE cells. Finally, experiments in an experimental model of uveitis proved that local A20 overexpression plays a protective role in EAU which coincides with the maintenance of BRB integrity and inhibition of CD4+T cell activation.

## Data Availability Statement

The raw data supporting the conclusions of this article will be made available by the authors, without undue reservation.

## Ethics Statement

The studies involving human participants were reviewed and approved by The First Affiliated Hospital of Chongqing Medical University. The patients/participants provided their written informed consent to participate in this study. The animal study was reviewed and approved by The First Affiliated Hospital of Chongqing Medical University.

## Author Contributions

JH and HL designed the experiments, interpreted the data, and wrote the manuscript. JH, SY, and XH performed experiments. JH, CW, YZ, JT, JY, and LY interpreted the data. JH and HL edited the manuscript. All authors contributed to the article and approved the submitted version.

## Funding

This study was supported by the National Natural Science Foundation Project (81770913, 81974131, 81900887).

## Conflict of Interest

The authors declare that the research was conducted in the absence of any commercial or financial relationships that could be construed as a potential conflict of interest.
