# Supplementary material for: A20 Inhibits Intraocular Inflammation in Mice by Regulating the Function of CD4+T Cells and RPE Cells
Source: Front Immunol. 2021 Feb 4;11:603939. doi: 10.3389/fimmu.2020.603939 (PMC7890008; doi:10.3389/fimmu.2020.603939)
Supplement: Supplementary file 1 [file DataSheet_1.docx]

| Gene | Forward | Reverse |
| --- | --- | --- |
| m IL-17 | 5’-CTCAACCGTTCCACGTCACCCT-3’ | 5’-CCAGCTTTCCCTCCGCATT-3’ |
| m IFN-γ | 5’-TCAAGTGGCATAGATGTGGAAGAA-3’ | 5’-TGGCTCTGCAGGATTTTCATG-3’ |
| m MCP-1 | 5’-TTAAAAACCTGGATCGGAACCAA-3’ | 5’-GCATTAGCTTCAGATTTACGGGT-3’ |
| m TNF-α | 5’-ACCCTGGTATGAGCCCATATAC-3’ | 5’-ACACCCATTCCCTTCACAGAG-3’ |
| m A20 | 5’-CAGTGGGAAGGGACACAACT-3’ | 5’-GCAGTGGCAGAAACTTCCTC-3’ |
| m IL-6 | 5’-GAGGATACCACTCCCAACAGACC-3’ | 5’- AAGTGCATCATCGTTGTTCATACA-3’ |
| m GAPDH | 5’-ACCCAGAAGACTGTGGATGG-3’ | 5’-CACATTGGGGGTAGGAACAC-3’ |
| m T-bet | 5’-CCAGTATCCTGTTCCCAGCC-3’ | 5’-CATAACTGTGTTCCCGAGGTGTC-3’ |
| m RORC | 5’-CCGCTGAGAGGGCTTCAC-3’ | 5’-TGCAGGAGTAGGCCACATTACA-3’ |
| m Foxp3 | 5’-CCAGTATCCTGTTCCCAGCC-3’ | 5’-CATAACTGTGTTCCCGAGGTGTC-3’ |
| m GATA3 | 5’-TGGCGCCGTCTTGATAGTTT-3’ | 5’-GCTCAGAGACGGTTGCTCTT-3’ |
| h β-actin | 5’-GAGAAAATCTGGCACCACACC-3’ | 5’-GGATAGCACAGCCTGGATAGCAA-3’ |
| h A20 | 5’-CGTCCAGGTTCCAGAACACCATTC-3’ | 5’-TGCGCTGGCTCGATCTCAGTTG-3’ |
| h IL-17 | 5’-TAATGGCCCTGAGGAATGGC-3’ | 5’-AGGAAGCCTGAGTCTAGGGG-3’ |
| h IFN-γ | 5’- AGGCTTTATCTCAGGGGCCA-3’ | 5’-AGCACTGGCTCAGATTGCAG -3’ |
|  |  |  |

Supplementary Table 1. The primer used in this study

This study included 32 active BD patients (30 males and 2 females, average age: 34.91±8.014 years old), 14 inactive BD patients(12 males and 2 females, average age:37.43±7.583 years old) , 32 active VKH patients(18 males and 14 females , 37.56±8.347 years old), 17 inactive VKH patients (7 males and 10 females, average age:37.71±9.393 years old) and healthy controls (24 males and 22 females, average age: 35.67±7.888 years old)

Supplementary Table 2. Basic information of inactive VKH patients in this study.

| Number | Age | Sex | Medications |
| --- | --- | --- | --- |
| 1 | 30 | M | Prednisone15mg, qd, CsA 125mg, qd |
| 2 | 46 | M | Prednisone 20mg, qd, CsA 100mg, qd |
| 3 | 55 | F | Prednisone 5mg, qd, CsA 100mg, qd |
| 4 | 24 | F | Prednisone 5mg, qd, CsA 100mg, qd |
| 5 | 54 | F | Prednisone 20mg, qd, CsA 100mg, qd |
| 6 | 28 | F | Prednisone 5mg, qd, CsA 100mg, qd |
| 7 | 45 | F | Prednisone 15mg, qd, CsA 100mg, qd |
| 8 | 37 | M | Prednisone 5mg, qd, CsA 100mg, qd |
| 9 | 39 | F | Prednisone 20mg, qd, CsA 100mg, qd |
| 10 | 28 | F | Prednisone 20mg, qd, CsA 125mg, qd |
| 11 | 27 | F | Prednisone 5mg, qd, CsA 100mg, qd |
| 12 | 31 | M | Prednisone 5mg, qd, CsA 100mg, qd |
| 13 | 34 | M | Prednisone 5mg, qd, CsA 100mg, qd |
| 14 | 45 | F | Prednisone 20mg, qd, CsA 100mg, qd |
| 15 | 35 | F | Prednisone 15mg, qd, CsA 100mg, qd |
| 16 | 45 | M | Prednisone 15mg, qd, CsA 100mg, qd |
| 17 | 38 | M | Prednisone 5mg, qd, CsA 100mg, qd |
|  |  |  |  |

Supplementary Table 3. Basic information of inactive BD patients in this study.

| Number | Age | Sex | Medications |
| --- | --- | --- | --- |
| 1 | 42 | F | Prednisone15mg, qd, IFN-α: 3*10^7^ IU qd |
| 2 | 34 | M | Prednisone 15mg, qd, CsA 75mg, qd |
| 3 | 38 | M | Prednisone 5mg, qd, CsA 50mg, bid |
| 4 | 48 | M | Prednisone 10mg, qd, CsA 50mg, bid |
| 5 | 45 | M | Prednisone 5mg, qd, CsA 50mg, bid |
| 6 | 43 | M | Prednisone 15mg, qd, CsA 50mg, bid |
| 7 | 29 | M | Prednisone 15mg, qd, CsA 125mg, qd |
| 8 | 21 | M | Prednisone 10mg, qd, IFN-α: 3*10^7^ IU, qod |
| 9 | 40 | M | Prednisone 15mg, qd, CsA 50mg, qd |
| 10 | 37 | M | Prednisone 20mg, qd, CsA 125mg, qd |
| 11 | 27 | M | Prednisone 5mg, qd, CsA 75mg, qd |
| 12 | 38 | F | Prednisone 10mg, qd, CsA 50mg, bid |
| 13 | 45 | M | Prednisone 5mg, qd, CsA 50mg, bid |
| 14 | 37 | M | Prednisone 20mg, qd, CsA 75mg, qd |

Supplementary Table 4. Basic information of active BD patients in this study.

| Number | Age | Sex | Medications |
| --- | --- | --- | --- |
| 1 | 34 | M | None |
| 2 | 30 | M | None |
| 3 | 38 | M | None |
| 4 | 30 | M | None |
| 5 | 40 | M | None |
| 6 | 46 | M | None |
| 7 | 28 | M | None |
| 8 | 25 | M | None |
| 9 | 35 | M | None |
| 10 | 52 | F | None |
| 11 | 31 | F | Without medication for 3 months |
| 12 | 27 | M | Prednisone 20mg, qd, CsA 125mg, qd, |
| 13 | 35 | M | Without medication for 12 months |
| 14 | 32 | M | None |
| 15 | 42 | M | None |
| 16 | 32 | M | Without medication for 2 months |
| 17 | 43 | M | None |
| 18 | 56 | M | Prednisone 20mg, qd, CsA 125mg, qd, |
| 19 | 27 | M | Prednisone 20mg, qd, CsA 75mg, qd, CTX 50mg, qd |
| 20 | 33 | M | Prednisone 25mg, qd, CsA 75mg, qd, CTX 50mg, qd |
| 21 | 30 | M | Prednisone 25mg, qd, CsA 75mg, qd, CTX 50mg, qd |
| 22 | 32 | M | Prednisone 5mg, qd |
| 23 | 32 | M | Prednisone 20mg, qd, CsA 100mg, qd |
| 24 | 27 | M | Prednisone 20mg, qd, CsA 125mg, qd |
| 25 | 28 | M | Prednisone 20mg, qd |
| 26 | 38 | M | Prednisone 20mg, qd, CTX 50mg, qd |
| 27 | 20 | M | Prednisone 20mg, qd, CTX 50mg, qd |
| 28 | 29 | M | Prednisone 10mg, qd, CsA 50mg, qd |
| 29 | 39 | M | Prednisone 20mg, qd, CsA 50mg, qd |
| 30 | 45 | M | Prednisone 5mg, qd, CsA 125mg, qd |
| 31 | 44 | M | Prednisone 10mg, qd |
| 32 | 37 | M | Prednisone 20mg, qd, CsA 50mg, qd |

**Supplementary Figures
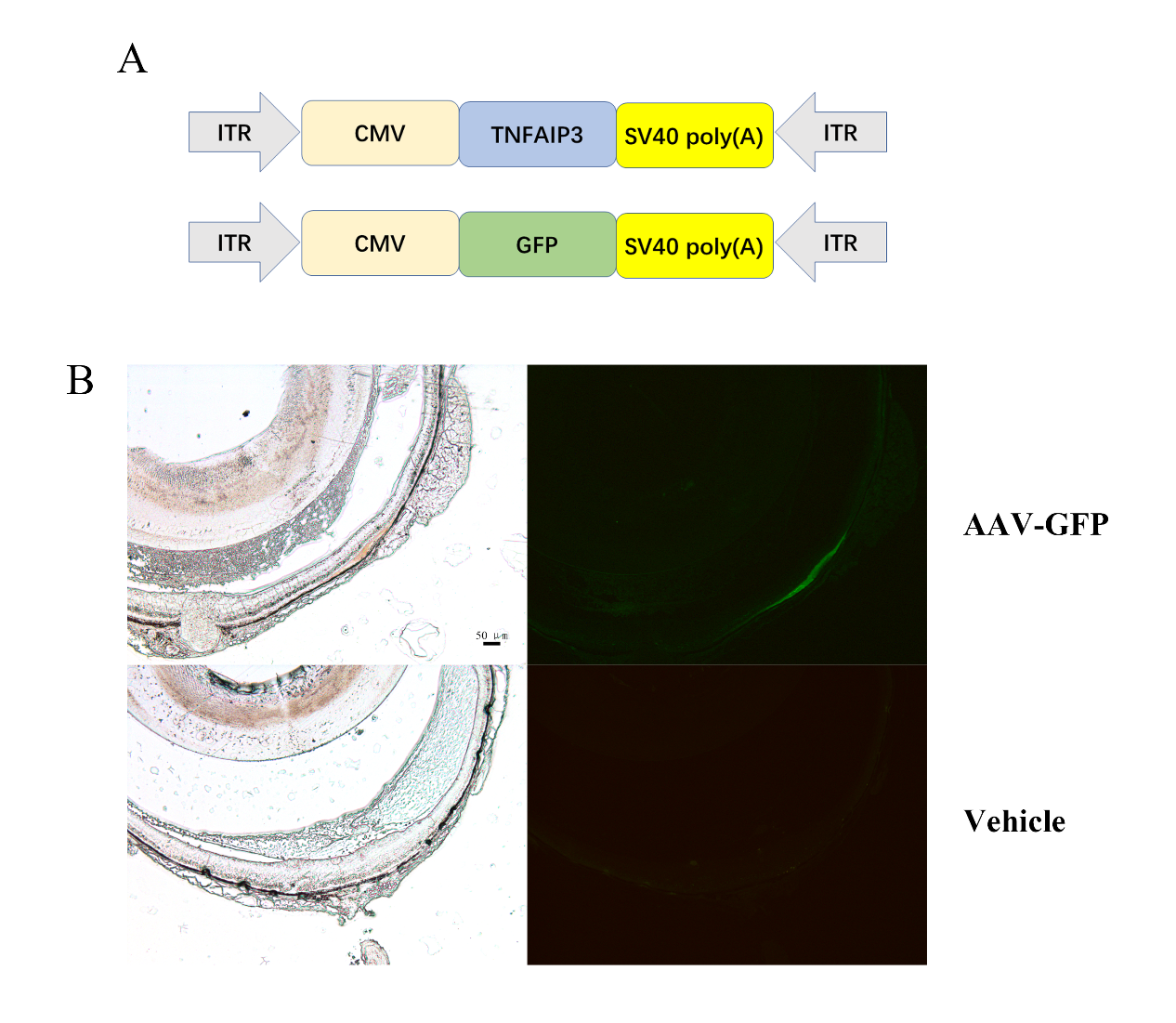
**

**Fig.S1 Details of AAV-TNFAIP3 and AAV-GFP. A.** Schematic representation of the AAV vectors carrying gene *TNFAIP3* and *GFP*. **B**. The expression of GFP in a cross section of the mouse eye after injecting *AAV-GFP*.


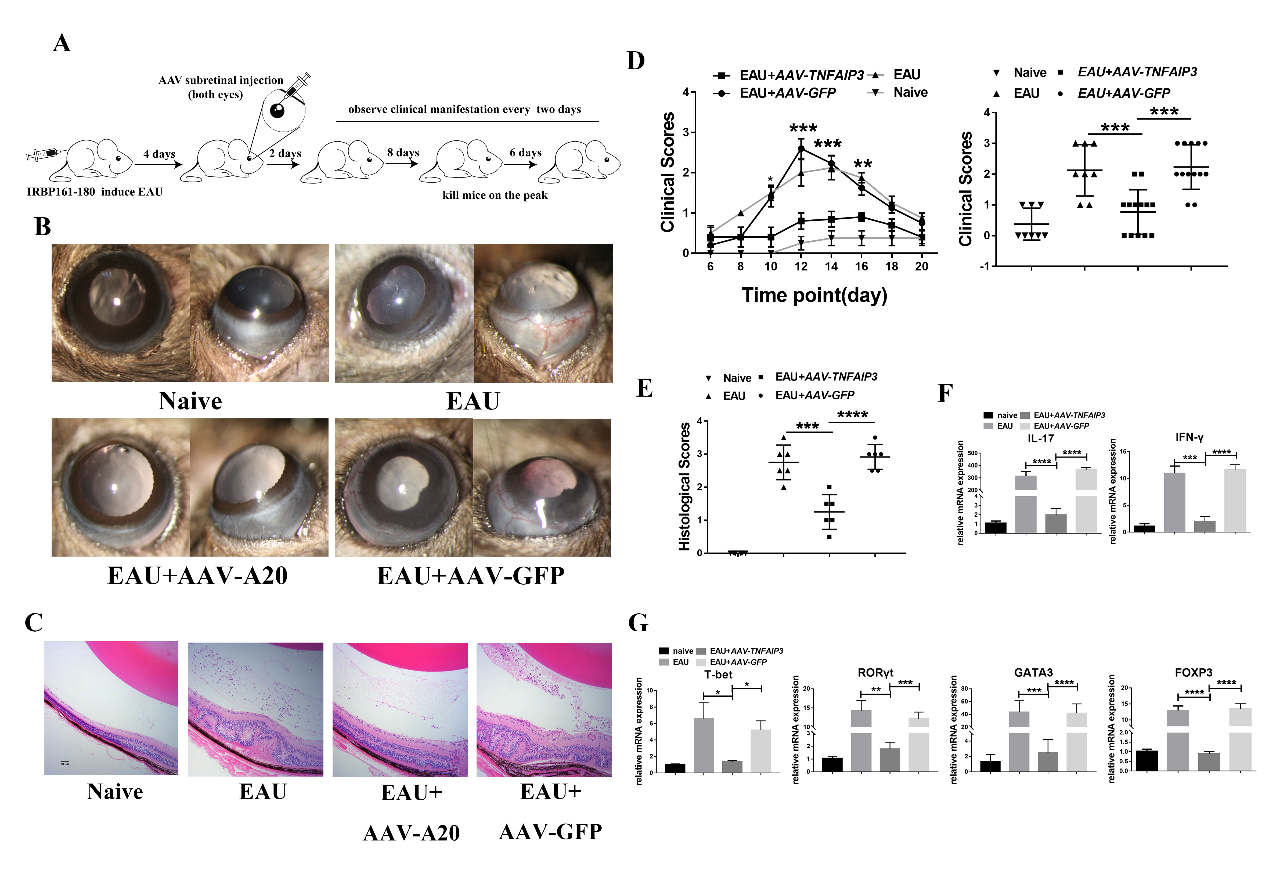


**Fig.S2 A20 overexpression on day 4 after IRBP immunization mainly inhibited activation of Th1 and Th17 cells. A.** Both eyes were injected with *AAV-TNFAIP3* or *AAV-GFP* on the 4^th^ day after inducing EAU. **B** Representative slit-lamp images of EAU mice at the 14^th^ day after immunization from the frontal and lateral view. **C.** The representative histological image of eyes harvested at the 14th day after immunization. **D.** The clinical scores were assessed every two days from day 6 after inducing EAU. A separate panel shows the clinical score at the peak of EAU (day 14) (n≥6 per group, p value was compared between the AAV-TNFAIP3 group and AAV-GFP group). **E.** Histological scores were assessed by hematoxylin and eosin (H&E) staining of paraffin-embedded sections. **F.** Relative mRNA expression of Th1 and Th17 cytokines IFN-γ and IL-17 by RT-qPCR (n≥4 per group). **G.** Relative mRNA expression of transcription factors T-bet, RORγT, GATA3 and FOXP3 in CD4+T cells by RT-qPCR (n≥4 per group). Scale bar = 50 μ m. Data are shown as mean ± SD. One-way ANOVA was used, ns p>0.05, **p<0.01, ***p<0.001, ****p<0.0001.


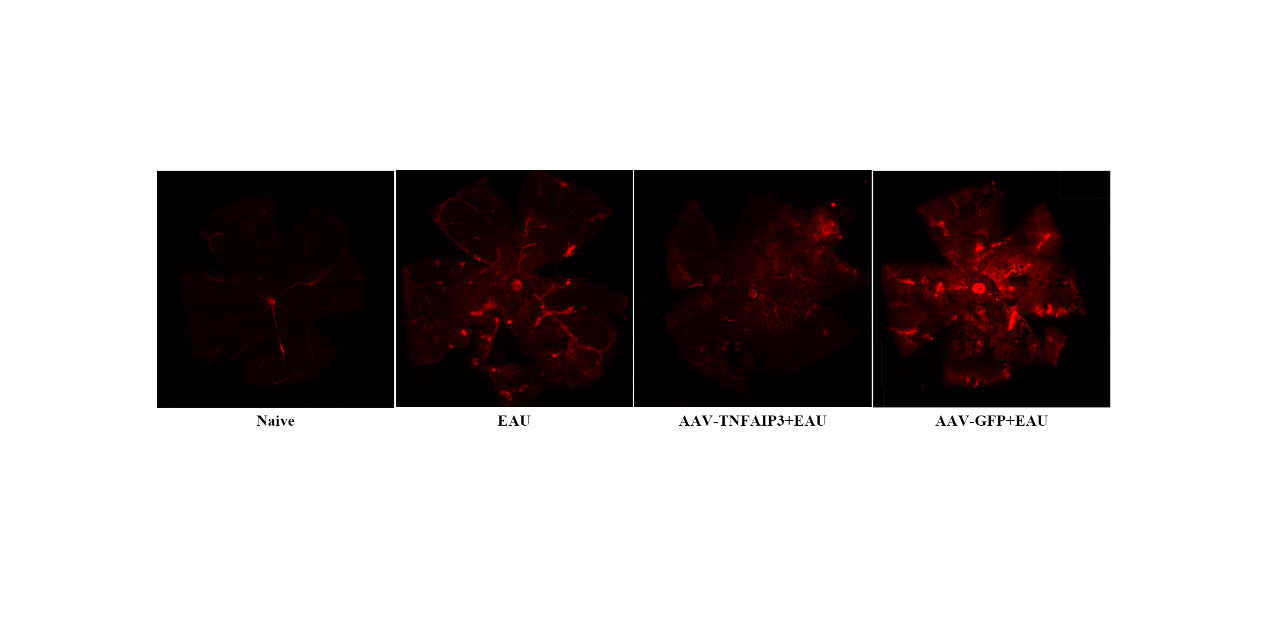


**Fig S3. A20 overexpression on day 4 after IRBP immunization had no effect on protecting blood-retinal-barrier (BRB) integrity.** Representative Evans-blue image of retinas from EAU mice in which A20 overexpression was induced 4 days after IRBP immunization.


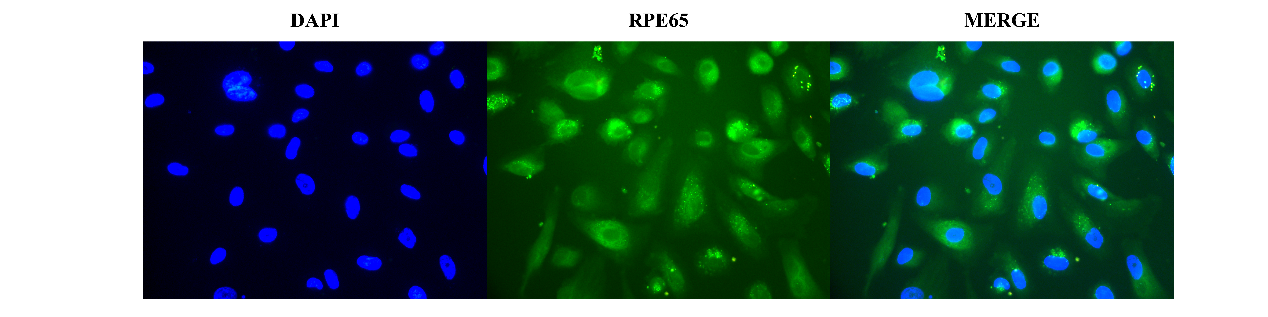


**Figure S4. Identification of cultured hRPE cells by analysis of RPE65 expression.** The ratio of RPE65-positive cells was calculated as the percentage of RPE65-positive cells in the DAPI-positive cells. The ratio was >90%. The scale bar 100 μm, magnification ×200.
